# Deep learning–assisted, pathogenesis-informed lung histopathology scoring in preclinical mouse models of SARS-CoV-2 and influenza A infection

**DOI:** 10.3389/fimmu.2026.1826608

**Published:** 2026-05-20

**Authors:** Hyeok-Won An, Hyeon Ah Kim, Tae Hun Ha, Sang Hyeok Seok, Yu Jin Lee, Na Yun Lee, Gyeong Min Yoon, Yang-Kyu Choi, Ho Young Lee, Won Gi Yoo, Je Kyung Seong, Jun Won Park

**Affiliations:** 1 Department of Laboratory Animal Medicine, College of Veterinary Medicine, Seoul National University, Seoul, Republic of Korea; 2 Department of Laboratory Animal Medicine, College of Veterinary Medicine, Konkuk University, Seoul, Republic of Korea; 3 Department of Nuclear Medicine, Seoul National University Bundang Hospital, Seongnam, Republic of Korea; 4 Laboratory of Veterinary Parasitology, College of Veterinary Medicine, Seoul National University, Seoul, Republic of Korea; 5 Laboratory of Developmental Biology and Genomics, Research Institute for Veterinary Science, and BK21 PLUS Program for Creative Veterinary Science Research, College of Veterinary Medicine, Seoul National University, Seoul, Republic of Korea; 6 Korea Model animal Priority Center, Seoul National University, Seoul, Republic of Korea; 7 Interdisciplinary Program in Bioinformatics and BIO MAX/N-Bio Institute, Seoul National University, Seoul, Republic of Korea; 8 Interdisciplinary Program of Cancer Biology, Seoul National University Cancer Research Institute, Seoul, Republic of Korea

**Keywords:** convolutional neural network, deep learning, histopathology, inflammation, mouse

## Abstract

**Introduction:**

SARS-CoV-2 and influenza A virus (IAV) cause viral pneumonia, yet their lung lesions evolve with distinct spatial organization and resolution-phase architecture. In preclinical murine studies, H&E histopathology is a primary endpoint, but burden-focused semiquantitative scoring can miss pathogen- and phase-specific differences in lesion topology, compartmental involvement, inflammatory organization, and repair. We aimed to define virus- and phase-specific morphologic signatures and translate them into a practical, pathogenesis-informed scoring guide, supported by whole-slide convolutional neural network (CNN) analysis with class activation mapping (CAM).

**Methods:**

Mice were infected under standardized conditions and evaluated during the early, peak-injury, and late phases of infection, corresponding to 2~3, 5~8, and 14 days post-infection (dpi), respectively. Lungs were assessed by H&E with semiquantitative scoring and by immunostaining to map viral antigen distribution and epithelial tropism. Whole-slide CNN models were trained for virus- and phase-specific classification, and CAM localized discriminative regions.

**Results:**

Dose titration established reproducible lethal and sublethal infection conditions for both viruses. Viral antigen kinetics diverged, with SARS-CoV-2 peaking early and declining toward clearance by the resolution phase, whereas IAV peaked later and declined by the resolution phase, paralleling distinct injury–repair trajectories. CNN/CAM analysis distinguished virus- and phase-specific histologic patterns across the early, peak-injury, and resolution phases of infection and highlighted spatial signatures consistent with expert review. At the peak-injury phase, SARS-CoV-2 lungs showed broad alveolar/interstitial involvement, whereas IAV exhibited bronchocentric inflammatory organization. During the resolution phase, IAV showed prominent epithelial regeneration with remodeling-forward architecture, while SARS-CoV-2 more often retained localized residual inflammatory foci. Across both infections, tissue inflammatory composition shifted over time, with higher neutrophil representation during the peak-injury phase and a relative increase in lymphocytic representation during the resolution phase. Integrating lesion topology/distribution, edema, epithelial injury–regeneration, remodeling features, and lymphocyte predominance, we proposed a pathogen-resolved, phase-informed histopathology scoring guide with recommended evaluation windows for each model.

**Conclusion:**

Together, these findings define virus- and phase-specific morphologic programs that inform respiratory virus pathogenesis in mice and can be translated into practical scoring criteria for preclinical respiratory virus studies.

## 1 Introduction

Respiratory viral infections caused by SARS-CoV-2 and influenza A virus continue to impose substantial global health burdens (1–3). Although these viruses share early clinical features and can both progress to severe pneumonia and acute respiratory distress syndrome in high-risk populations, they differ in host cell entry mechanisms and in the dominant lung compartments affected during infection (4–6). These biological differences have shaped the development and use of preclinical models for studying pathogenesis and for evaluating candidate therapeutics and vaccines (7–9). For SARS-CoV-2, conventional wild-type mice show limited susceptibility because murine ACE2 does not efficiently support viral entry, and human ACE2-expressing mouse models have therefore been widely adopted (10–12). Among these, K18-hACE2 mice are extensively used because disease severity, lethality, and clinical course can be tuned by inoculation dose and vary with the infecting viral strain or variant (13, 14). In contrast, IAV readily infects commonly used inbred mouse strains, including BALB/c and C57BL/6 mice, although susceptibility and disease severity still vary with host genetic background, viral strain, and inoculation dose (15–17).

In these preclinical models, lung histopathology is a core analytical approach because it enables direct identification of lesion phenotypes and the affected cellular compartments, thereby providing essential context for interpreting infection-associated tissue injury (18, 19). Accordingly, semiquantitative histopathologic scoring of H&E-stained lung sections is commonly used to facilitate group-level comparisons in preclinical studies (18, 20, 21). In parallel, complementary descriptors that capture lesion distribution and compartmental predominance, together with principles that support reproducible scoring, can facilitate time-resolved interpretation of lung lesions across infection phases, and may be particularly informative in comparative designs across pathogens and time points (18, 19, 21). While conventional semiquantitative scoring provides a robust foundation for evaluating tissue injury, differentiating between pathogens that present with highly overlapping inflammatory profiles, such as SARS-CoV-2 and IAV, remains inherently challenging (18, 22). Standard inflammation scoring effectively captures global disease severity, but resolving subtle, virus-specific spatial lesion patterns and compartmental predominance often requires additional granularity. Furthermore, to complement expert visual interpretation and mitigate inherent observational variability, there is a growing need for objective computational approaches that can reproducibly capture complex morphological features across different infection phases, thereby augmenting traditional histopathologic evaluation (19, 23–25).

In the present study, we established matched lethal and sublethal infection conditions for SARS-CoV-2 and IAV in K18-hACE2 mice and performed a time-resolved comparative analysis of lung pathology across the early, peak-injury, and resolution phases of infection. By integrating CNN-based whole-slide image analysis with class activation mapping and expert histopathologic review, we asked whether virus- and phase-associated morphologic patterns can be resolved even when overall inflammatory burden overlaps, and whether these spatial features can be translated into pathogenesis-informed histopathology scoring criteria for preclinical respiratory virus studies.

## 2 Materials and methods

### 2.1 Mice

The mice (6~16 weeks old, male) used in these studies were obtained from the Jackson Laboratory (B6. Cg-Tg(K18-ACE2)2Prlman/J) and are congenic on the C57BL/6 background. All protocols were approved by the Institutional Animal Care and Use Committee of the Seoul National University Bundang Hospital (IACUC number BA-2008-301-071-05). The Ji Seok Young Research Centre is fully accredited by the Association for Assessment and Accreditation of Laboratory Animal Care. All animals were cared for in accordance with the Institute for Laboratory Animal Research Guide for the Care and Use of Laboratory Animals, eighth Edition. The Seoul National University Bundang Hospital Institutional Biosafety Committee approved the procedures for sample handling, inactivation, and transfer from animal biosafety level 3 (ABSL3) containment. Virus-inoculated mice were intranasally administered the indicated virus dose in a total volume of 50 μL DMEM, a volume within the range commonly used for lower respiratory tract delivery and murine respiratory virus infection models (13, 26–28). Vehicle-control mice were intranasally administered the same total volume of DMEM using the same intranasal administration procedure. Histopathologic review confirmed that vehicle-control lungs did not show appreciable acute procedure-associated lesions, including inflammatory infiltration, edema, hemorrhage, consolidation, or epithelial injury. The weight, temperature, and health of the mice were monitored daily and euthanized if pre-specified humane endpoint criteria were met, in accordance with the approved IACUC protocol. Mice were sacrificed in a CO2 chamber at predefined post-infection time windows selected to capture early, peak-injury, and resolution phase lung pathology, corresponding to 2~3, 5~8, and 14 dpi, respectively. All experiments with SARS-CoV-2 and Influenza A were performed in a BSL3 Laboratory at the Seoul National University Bundang Hospital.

### 2.2 Virus

The original Wuhan (WA1) strain of SARS-CoV-2 (accession number: NCCP43326/Korea) and Influenza A virus (H1/N1) (NCCP42467/Korea) were procured from the Korea Centers for Disease Control and Prevention (KDCDC03/2020), and Vero E6 cell (CRL-1586) was procured from the Korea Microbial Resource Centre (KCTC). Vero E6 was maintained in Dulbecco’s Modified Eagle Medium (DMEM; Life Technologies, CA, USA) containing 10% fetal bovine serum (FBS; Life Technologies). SARS-Cov-2 was inoculated with Vero E6 cells to confirm the cytopathic effect on the third day; virus titer was measured by plaque assay and stored at -70 °C.

### 2.3 Histologic processing, H&E staining

Lung tissues were fixed in neutral buffered 10% formalin for 1 day and processed for paraffin embedding using an automated tissue processor. Tissues were dehydrated through graded ethanol using Ethyl Alcohol 99.5% (Korea Alcohol Industrial Co., Ltd., Yongin, Republic of Korea) in a graded series (70%, 80%, 90%, 95%, and 100% ethanol three times; 50 min each at room temperature), cleared in xylene (Daejung Chemicals & Metals Co., Ltd., Siheung, Republic of Korea, 8587-4410) through three changes of 50 min each at room temperature, and infiltrated with paraffin (Sigma-Aldrich, St. Louis, MO, USA, 1.15161.2504) through four changes of 50 min each at 60 °C. Vacuum-assisted processing was applied during the dehydration and xylene clearing steps and during the last three paraffin infiltration steps, whereas the first paraffin step was performed without vacuum. All processing steps were performed at slow mixing speed.

Formalin-fixed, paraffin-embedded lung sections (3 μm) were stained with hematoxylin and eosin (H&E). Briefly, sections were deparaffinized in xylene (two changes, 3 min each), rehydrated through graded ethanol (100% ethanol, two changes, 3 min each; 95% ethanol, 3 min; 90% ethanol, 3 min; 70% ethanol, 3 min), and rinsed in distilled water for 5 min. Slides were stained with Harris hematoxylin (Harris Hematoxylin, YD Diagnostics Corp., Yongin, Republic of Korea, S2-5) for 5min, washed in water for 5 min, differentiated in 0.1% hydrochloric acid solution prepared from 1 N hydrochloric acid (Samchun Pure Chemical Co., Ltd., Pyeongtaek, Republic of Korea, 7647-01-0) for 25 s, and washed again in water for 5 min. Sections were then immersed in 95% ethanol for 1 min and counterstained with eosin-phloxine solution (Labcore Co., Ltd., Seoul, Republic of Korea, 051614-500) for 5 min. After staining, slides were dehydrated in 95% ethanol for 5 sec, followed by 100% ethanol twice for 3 min each, cleared in xylene for 3 min, and kept in xylene until mounting.

### 2.4 Histopathologic evaluation and semiquantitative scoring

Histologic evaluation and manual histopathological scoring of H&E-stained lung sections were performed by a veterinary pathologist (J.W. Park) who was blinded to group allocation. Scoring was conducted using digitized whole-slide images and was based on whole-slide/lobe-level assessment rather than predefined field-based sampling. Because the aim of this study was to capture the overall burden and spatial distribution of lung lesions, a fixed number of microscopic fields per mouse was not used. Instead, all lung lobes present on each section were systematically reviewed and integrated into a single composite score without preferential selection of any specific lobe. Lesion distribution and overall extent were initially assessed at low magnification (e.g., ×2–×4), followed by evaluation of specific histopathologic features at higher magnification (e.g., ×10–×40) as needed. This approach was used to minimize field-selection bias and to ensure comprehensive assessment of lesion heterogeneity across the entire section.

Inflammation was graded on a 0–5 scale according to the estimated percentage of lung parenchyma involved by inflammatory cell infiltrates within alveolar septa, alveolar spaces, peribronchiolar regions, and/or perivascular areas. A score of 0 indicated no observable inflammatory cell infiltration. A score of 1 (minimal) was assigned when scattered inflammatory cells involved less than 10% of the examined area. A score of 2 (mild) corresponded to focal but more consistent infiltration involving approximately 11–25% of the lung tissue. A score of 3 (moderate) represented multifocal infiltrates involving 26–50% of the lung parenchyma. A score of 4 (severe) indicated widespread inflammatory infiltration affecting 51–75% of the examined area. A score of 5 (markedly severe) was assigned when diffuse infiltration involved more than 75% of the lung tissue and was frequently accompanied by broad areas of consolidation. Although inflammatory infiltrates were frequently distributed in alveolar, bronchocentric, or perivascular patterns, grading was based on total affected area rather than distribution pattern. All scoring was performed using predefined criteria, and slide review was conducted blinded to experimental group allocation.

### 2.5 Immunohistochemistry and digital analyses

The replicate paraffin sections were dewaxed, rehydrated, and subjected to antigen retrieval by heating at 100 °C for 20 minutes in 0.01 M citrate buffer (pH 6.0). The ImmPRESS Peroxidase Polymer kit (Vector Laboratories, Burlingame, CA) was used for immunostaining in accordance with the manufacturer’s protocol. Briefly, the slides were incubated with 2.5% horse serum, goat serum for anti- and blocking and then incubated for 30 minutes at room temperature with the primary antibodies. Rabbit anti-SARS-CoV-2 nucleocapsid (Genetex, GTX635679, RRID: AB_2888553, CA, USA), rabbit anti-Influenza A nucleoprotein (Genetex, GTX125989, RRID: AB_11168364), goat anti-SP-C (Santa Cruz Biotechnology, SC-13979, RRID: AB_2185502, Texas, USA) were used as primary antibodies. After washing, the slides were incubated for 30 minutes with the appropriate peroxidase polymer-linked secondary antibodies. The slides were subjected to colorimetric detection with the ImmPact DAB substrate (SK-4105, Vector Laboratories, RRID: AB_2336520, CA, USA). The slides were counterstained with Meyer’s hematoxylin for 10 seconds. Negative controls were performed by omitting the primary antibody and substituting it with diluent.

For digital pathology, IHC slides were digitally scanned using Pannoramic SCAN slide scanner (3D HISTECH) at 40x magnification. IHC imaging quantifications were performed with digital image analysis (QuPath-0.5.1, University of Edinburgh, UK). The Positive Cell Detection function in QuPath, using the optical density (OD) sum channel, was used to detect SP-C DAB signals and quantify the number of positively stained cells. First, we adjusted the parameters in this algorithm to accurately match the manual count of stained cells by a pathologist (J.W. Park) with automated counts obtained from the tuned algorithms. The set of tuned parameters for each marker was saved and used to run analysis on each tissue.

### 2.6 TUNEL assay

The detection of apoptotic cells in tissue sections was performed using the terminal deoxynucleotidyl transferase dUTP nick end labeling (TUNEL) assay and the *in situ* Apoptosis Detection Kit (MK500; Takara Biotechnology, Dalian, China, RRID: AB_2800362), in accordance to the manufacturer’s instructions. TUNEL image quantification was performed using digital image analysis in QuPath (v0.5.1; University of Edinburgh, Edinburgh, UK) on digitized slide images. The Positive Cell Detection function in QuPath, using the optical density (OD) sum channel, was applied to detect TUNEL-positive signals and quantify the number of TUNEL-positive nuclei.

### 2.7 Immunofluorescence staining

Formalin-fixed paraffin-embedded (FFPE) slides were deparaffinized and sequentially rehydrated using ethanol. The slides were immersed in antigen retrieval solution (Dako) and incubated for antigen retrieval at high pressure using a cooker. After cooking, the slides were incubated with 2.5% horse serum in PBS for blocking and then incubated overnight at 4 °C with the primary antibodies. Mouse anti-SARS-CoV-2 nucleocapsid (Sino Biological, 40143-MM08, RRID: AB_2827978, Beijing, China), rabbit anti-PDPN (Santa Cruz Biotechnology, sc-134483, RRID: AB_2268064), goat anti-SP-C (Santa Cruz Biotechnology, sc-13979, RRID: AB_2185502), rabbit anti-cleaved caspase3 (Cell signaling, 9664S, RRID: AB_2070042, MA, USA), rabbit anti-Influenza A virus Nucleoprotein (Genetex, GTX125989, RRID: AB_11168364) and goat anti-influenza A H1N1 (Genetex, GTX40841, RRID: AB_424290) were used as primary antibodies. Each primary antibody derived from diverse species was detected with the use of Alexa488- (Invitrogen, MA, USA), Alexa568- (Invitrogen, MA, USA) conjugated secondary antibodies. Nuclear staining was performed with the use of DAPI (4′,6-Diamidino-2-phenylindole dihydrochloride) (Sigma).

### 2.8 Imaging and microscopy

IHC slides were digitally scanned at ×40 magnification using a Pannoramic 250 Flash III slide scanner (3DHISTECH, Budapest, Hungary). Whole-slide images were reviewed using CaseViewer software (3DHISTECH). Since IHC slides were digitized by brightfield whole-slide scanning, laser settings were not applicable.

Immunofluorescence images were acquired using a ZEISS LSM 800 confocal laser scanning microscope (Carl Zeiss, Germany). Tissue sections were cut at 3 μm thickness. The microscope was equipped with 10× air (NA 0.30), 20× air (NA 0.80), 40× water immersion (NA 1.20), and 63× oil immersion (NA 1.40) objective lenses. The confocal system was configured with 405, 488, 561, and 633 nm laser lines, and image acquisition was performed using ZEISS ZEN software.

### 2.9 Convolutional neural network–based whole-slide image analysis

#### 2.9.1 Image dataset and labeling

Whole-slide H&E images of formalin-fixed, paraffin-embedded (FFPE) lung tissues were acquired from 153 mice (no infection [n = 35], SARS-CoV-2–infected [n = 81], and influenza A virus [IAV]–infected [n = 37]) derived from 13 preclinical trials (**Table 1**). For each animal, one representative H&E-stained section was obtained from each lung lobe and digitized as a whole-slide image (WSI). One WSI per lobe was used for subsequent analysis. All images were reviewed and assigned ground-truth labels for (i) virus group and (ii) infection phase. Infection phase was categorized into early, peak-injury, and resolution, corresponding to 2~3, 5~8, and 14 dpi, respectively. Accordingly, each image was labeled as one of seven categories: normal lung, SARS-CoV-2 early phase, SARS-CoV-2 peak-injury phase, SARS-CoV-2 resolution phase, IAV early phase, IAV peak-injury phase, or IAV resolution phase. This phase-aware labeling strategy was adopted to enable classification not only by pathogen identity but also by stage-dependent histomorphologic evolution.

**Table 1:** Information on the preclinical mouse cohorts used for SARS-CoV-2 and influenza A virus infection studies.

Cohort	Group	Mouse number	DPI	Infection dose (PFU)
1	Vehicle	4	7	–
	SARS-CoV-2	4	7	1×10^5^
2	SARS-CoV-2	4	3	1×10^5^
	SARS-CoV-2	4	7	1×10^5^
3	SARS-CoV-2	3	2	1×10^4^
	SARS-CoV-2	5	6~7	1×10^4^
4	SARS-CoV-2	4	6~7	1×10^5^
5	SARS-CoV-2	2	2	1×10^5^
	SARS-CoV-2	5	6~7	1×10^5^
6	SARS-CoV-2	2	2	1×10^4^
	SARS-CoV-2	5	6~7	1×10^4^
7	Influenza A	11	5~8	1×10^2~4^
8	Vehicle	3	7	–
9	Vehicle	4	7	–
10	Vehicle	3	14	–
	Influenza A	11	14	0.5~1x10^1^
11	Vehicle	4	7	1×10^5^
	SARS-CoV-2	5	2	
	SARS-CoV-2	3	7	
12	Vehicle	5	14	–
	Influenza A	5	2	1x10^1^
	Influenza A	5	7	1x10^1^
	Influenza A	5	14	1x10^1^
13	Vehicle	12	14	–
	SARS-CoV-2	12	2	1×10^2^
	SARS-CoV-2	11	7	1×10^2^
	SARS-CoV-2	12	14	1×10^2^

#### 2.9.2 Deep learning platform and classification models

All lobe-level images were imported into Neuro-T Pro software (Neuro-T Pro v4.4.1; Neurocle Inc., TissenBioFarm, Seoul, Korea) for supervised deep learning (DL)–based classification. Neuro-T is a no-code DL platform that automatically optimizes network architecture and hyperparameters (29–31). The software was used as an automated model-building environment for supervised image classification. Therefore, only the training settings directly accessible and verifiable by the user were reported in the present study. DL was conducted on a custom-built workstation running Windows 10, equipped with an Intel^®^ Core i7-12700K CPU (12 cores, 3.6 GHz), 128 GB of random access memory (RAM), and an NVIDIA^®^ GeForce RTX 3090 D6 graphics processing unit (GPU, 24 GB).

#### 2.9.3 Dataset partitioning and training strategy

The image dataset was partitioned using the software’s dataset management function into a training set (85%) and an independent test set (15%). The independent test set was held out from model training and used exclusively for final performance evaluation. During training, the platform’s built-in augmentation option was enabled to improve robustness against image-level variability and to reduce overfitting in the setting of a limited dataset. Because the present study aimed to assess whether routine H&E morphology contains sufficient information to resolve pathogen- and phase-specific lung injury patterns, classification models were trained using image-level class labels without manual lesion annotation. Thus, the network learned discriminative morphologic features directly from labeled lobe-level WSIs.

#### 2.9.4 Task-specific model development

For the classification analyses, separate CNN models were generated for five tasks: (1) 7-class classification, (2) peak-phase cross-virus classification, (3) resolution-phase cross-virus classification, (4) SARS-CoV-2 temporal classification, and (5) IAV temporal classification. The 7-class model distinguished normal lung, SARS-CoV-2 early-, peak-injury-, and resolution-phase images, and IAV early-, peak-injury-, and resolution-phase images. The peak-phase and resolution-phase cross-virus models compared SARS-CoV-2 and IAV within the same phase, whereas the SARS-CoV-2 temporal and IAV temporal models distinguished phase-specific morphologic changes within each virus. This task-specific design was intended to separately evaluate global multi-class performance, virus discrimination within matched phases, and temporal discrimination within each virus.

#### 2.9.5 Input image optimization and ablation analysis

An ablation study was performed to evaluate the effects of input image size and data augmentation settings across the classification tasks. As summarized in **Supplementary Table 1**, the 512 × 512 input setting with linear resizing and augmentation showed the most consistent overall performance across tasks and was therefore selected as the final configuration for model training. Accordingly, user-accessible training parameters of the best-performing model for each task, including batch size, number of epochs, number of layers, and initial learning rate, are summarized in **Supplementary Table 2**.

#### 2.9.6 Class activation map generation

CAMs were generated using the software’s built-in implementation to visualize image regions contributing to classification decisions and were used to support interpretability of morphology-based predictions. CAMs were reviewed by a board-certified veterinary pathologist (J.W. Park) to determine whether the network preferentially focused on histologically meaningful regions, such as inflammatory foci, peribronchial or perivascular lesions, alveolar damage, and remodeling-related structural alterations.

#### 2.9.7 Model performance evaluation

Model performance was evaluated on the independent test set using accuracy, precision, recall, and F1 score and confusion matrix analysis. Accuracy represents the proportion of correctly classified images among all evaluated images. Precision reflects the proportion of images predicted to belong to a given class that were truly assigned to that class, thereby indicating how reliable positive predictions were. Recall (also referred to as sensitivity) represents the proportion of images belonging to a given class that were correctly identified by the model, reflecting the model’s ability to capture true positive cases. The F1 score is the harmonic mean of precision and recall and provides a balanced measure of model performance, particularly when class distributions are unequal. In addition, a confusion matrix was constructed to compare the predicted and true class labels for each WSI, allowing visualization of class-specific correct classifications and misclassification patterns. For each task, confusion matrix analysis was used to identify which categories were readily separable and which showed greater morphologic overlap. These findings were interpreted together with CAM results to assess not only predictive performance but also the morphologic plausibility of the learned classification patterns.

### 2.10 Statistics

Statistical analyses were performed with the GraphPad Prism version 8 software. Semiquantitative scoring data were treated as ordinal variables and analyzed using the Kruskal–Wallis test followed by Dunn’s multiple-comparisons test. For comparisons of continuous variables between two independent groups, an unpaired two-tailed Student’s t-test was used when appropriate. The statistical test used for each comparison is indicated in the corresponding figure legend. Error bars indicate the standard deviations of the mean. Significance is indicated as follows: *p ≦ 0.05, **p ≦ 0.01, and ***p ≦ 0.001.

## 3 Result

### 3.1 Establishment of SARS-CoV-2 and IAV lethal and sublethal models

To establish conditions for pathological comparison, we generated both uniformly lethal (100% mortality) and sublethal (~70% mortality) infection models of SARS-CoV-2 and IAV in mice by titrating the inoculation dose. Lethal outcomes were defined as moribund status determined primarily by ≥15% body weight loss and severe vitality reduction.

**Figure 1:**
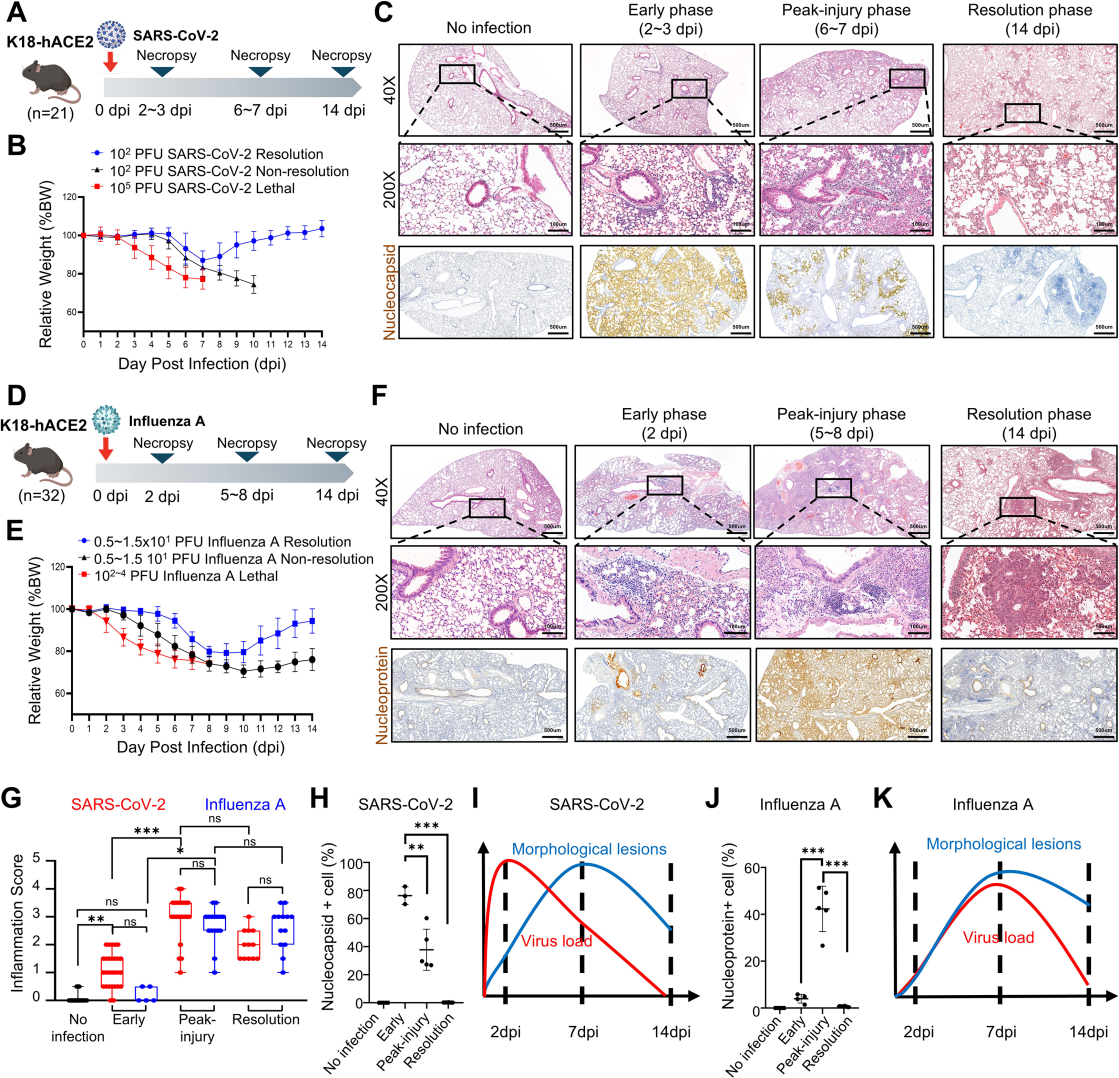
Establishment of infection conditions and time-resolved comparison of lung pathology between SARS-CoV-2 and Influenza A in K18-hACE2 mice. **(A)** Experimental design for SARS-CoV-2 infection in male K18-hACE2 mice. Mice were intranasally inoculated at the indicated doses and necropsied at the designated time points. **(B)** Body weight was monitored daily, and relative body weight (BW) was calculated as BW at the indicated dpi/BW at 0 dpi (%). **(C)** Representative lung sections from SARS-CoV-2–infected mice. H&E-stained sections are shown at the indicated time points, with boxed areas enlarged in the middle panels. Immunohistochemistry for SARS-CoV-2 nucleocapsid protein is shown in the bottom panels. Scale bars indicate 500 μm in low-magnification images and 100 μm in high-magnification images. **(D)** Experimental design for influenza A virus infection in male K18-hACE2 mice. Mice were intranasally inoculated at the indicated doses and necropsied at the designated time points. **(E)** Body weight monitoring and relative BW calculation were performed as in **(B)**. **(F)** Representative lung sections from influenza A virus–infected mice. H&E-stained sections are shown at the indicated time points, with boxed areas enlarged in the middle panels. Immunohistochemistry for influenza A nucleoprotein is shown in the bottom panels. Scale bars indicate 500 μm in low-magnification images and 100 μm in high-magnification images. **(G)** Semi-quantitative histopathologic inflammation scores at the indicated time points. Histopathologic scores were treated as ordinal variables and analyzed using the Kruskal–Wallis test followed by Dunn’s multiple-comparison test. Box-and-whisker plots show the median (center line), interquartile range (box), and min–max (whiskers). Group sizes were n = 32 (no infection; black dots), n = 28 (early phase; 2~3 dpi; red dots), n = 23 (peak-injury phase; 6~7 dpi; red dots), and n = 12 (resolution phase; 14 dpi; red dots) for SARS-CoV-2 (**Figure 1G**; red dots), and n = 5 (early phase; 2 dpi; blue dots), n = 16 (peak-injury phase; 5~8 dpi; blue dots), and n = 13 (resolution phase; 14 dpi; blue dots) for IAV (**Figure 1G**; blue dots). Significance is indicated as ns (not significant), *P < 0.05, **P < 0.01, and ***P < 0.001. **(H, J)** Quantification of viral antigen-positive cells in lung sections at the indicated time points. Each dot represents one mouse; group sizes were n = 5 (no infection), n = 3 (early phase), n = 5 (peak-injury phase), and n = 6 (resolution phase) for SARS-CoV-2 (**Figure 1H**), and n = 5 (no infection), n = 5 (early phase), n = 5 (peak-injury phase), and n = 6 (resolution phase) for IAV (**Figure 1J**). Significance is indicated as ns (not significant), *P < 0.05, **P < 0.01, and ***P < 0.001. **(I, K)** Schematic summary of the temporal relationship between viral load and morphologic lung lesions. Dashed lines indicate 2, 7, and 14 dpi.

Intranasal inoculation with Wuhan SARS-CoV-2 at 1×10^5^ PFU caused progressive weight loss and uniformly lethal outcomes during the peak-injury phase, whereas administration of 1×10² PFU resulted in approximately 70% mortality, with the remaining animals recovering body weight (**Figures 1A, B**). SARS-CoV-2–infected mice showed mild edema and limited immune cell infiltration during the early phase, followed by pronounced pneumonia during the peak-injury phase characterized by dense inflammatory cell accumulation and loss of normal airspaces, and exhibited more localized residual lesions during the resolution phase in surviving mice (**Figure 1C**).

For IAV, intranasal infection with 1×10²–1×10^4^ PFU resulted in rapid weight loss, and all mice became moribund by the peak-injury phase, whereas administration of 0.5–1×10¹ PFU produced approximately 70% lethality (**Figures 1D, E**). IAV-infected mice showed minimal histologic abnormalities during the early phase, progressed to severe pneumonia with prominent inflammatory cell accumulation and airspace loss during the peak-injury phase, and exhibited more localized residual lesions during the resolution phase in surviving mice (**Figure 1F**).

SARS-CoV-2–infected mice showed an increase in pulmonary inflammation from the early phase to the peak-injury phase by semi-quantitative histopathologic scoring, and inflammation scores were reduced during the late phase compared with the peak-injury phase (**Figure 1G**). In contrast, immunohistochemical analysis demonstrated abundant viral antigen during the early phase, followed by a reduction during the peak-injury phase and near-complete clearance during the resolution phase (**Figures 1C, H, I**). Collectively, this trajectory indicates a temporal offset between viral antigen burden and morphologic inflammation in SARS-CoV-2 infection, in which antigen burden peaks during the early phase whereas morphologic lesions become most prominent during the peak-injury phase (**Figures 1H, I**).

In IAV-infected mice, pulmonary inflammation similarly increased from the early phase to the peak-injury phase, but inflammation scores remained high during the resolution phase without a clear reduction relative to the peak-injury phase (**Figure 1G**). Immunohistochemical analysis showed weak, multifocal influenza nucleoprotein positivity during the early phase, peak antigen expression during the peak-injury phase, and a decline to near-complete clearance during the resolution phase, with only limited residual positivity (**Figures 1F, G**). This trajectory indicates a temporal dissociation between viral antigen burden and morphologic inflammation in IAV infection, with antigen expression peaking during the peak-injury phase and declining during the resolution phase, whereas inflammation remains elevated with persistent residual lesions during the resolution phase (**Figure 1J**).

Together, these data highlight distinct temporal relationships between viral antigen dynamics and morphologic lesion evolution in the SARS-CoV-2 and IAV mouse infection models (**Figures 1I, J**).

### 3.2 CNN-based classification distinguishes SARS-CoV-2 and influenza A lung histology across post-infection phase

**Figure 2:**
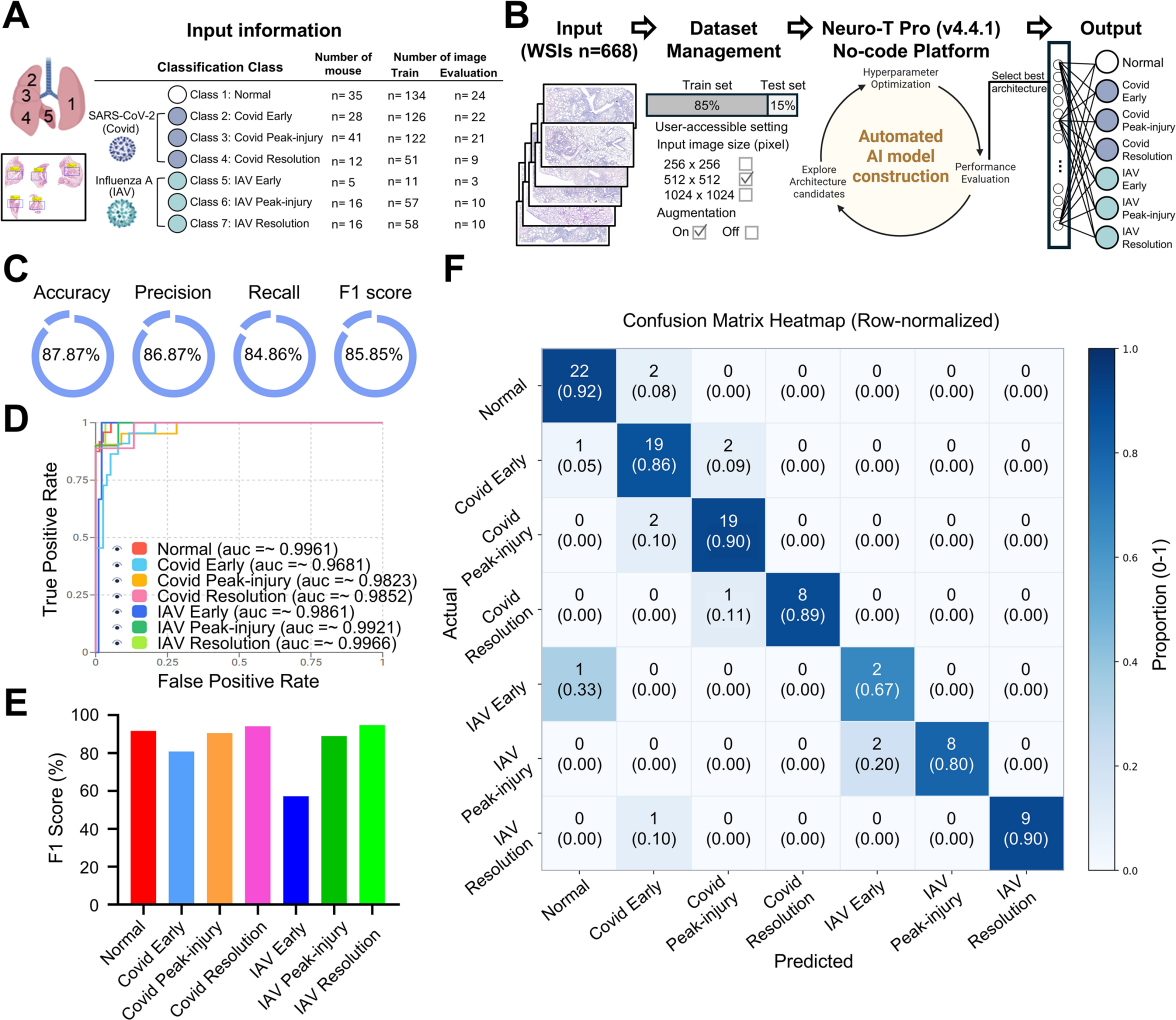
CNN-based multi-class classification of pathogen- and phase-specific lung histopathology in SARS-CoV-2 and Influenza A preclinical mouse models. **(A)** Input information for seven-class classification of H&E-stained lung whole-slide images (WSIs), including normal controls and SARS-CoV-2- and influenza A virus (IAV)-infected lungs in the early, peak-injury, and resolution phases. The numbers of mice and the training and evaluation images for each class are summarized in the table. **(B)** Overview of the CNN-based analysis pipeline. H&E-stained lung WSIs were used as input for deep learning–based feature learning and classification, and each WSI was assigned to one of seven output classes: normal, SARS-CoV-2 in the early, peak-injury, or resolution phase, or IAV in the early, peak-injury, or resolution phase. **(C)** Overall performance metrics of the seven-class classification model, including accuracy, precision, recall, and F1 score. **(D)** Receiver operating characteristic curves with area under the curve values for each of the seven classes. **(E)** Class-wise F1 scores for normal, SARS-CoV-2 in the early, peak-injury, or resolution phase, or IAV in the early, peak-injury, or resolution phase. **(F)** Row-normalized confusion matrix showing classification performance across the seven classes. In each cell, the upper number indicates the number of evaluation images, and the value in parentheses indicates the row-normalized proportion for the corresponding actual class.

Conventional semi-quantitative scoring captures overall inflammatory severity but is limited in resolving phase-associated disease states and spatial/morphologic heterogeneity from routine H&E sections. In particular, peak-injury and resolution lesions were not significantly separated by inflammation-burden scoring alone (**Figure 1G**), reflecting substantial overlap in overall inflammatory severity between these phases. This suggests that burden-based scoring may not fully capture phase-specific histologic organization. To address this limitation, we performed a CNN-based 7-class classification using lobe-level H&E images from normal controls and SARS-CoV-2– and IAV-infected mice across the early, peak-injury, and resolution phases of infection (**Figure 2A**). Model training and evaluation were conducted using Neuro-T (v4.4.1). When all five lung lobes were intact on a slide, one lobe-level overview image was generated per lobe from the whole-slide image (WSI), yielding five images per mouse. In total, 658 lobe-level images were collected, of which 559 were used for training and 99 were reserved as an independent test set (**Figures 2A, B**).

The 7-class model evaluation demonstrated high accuracy, precision, recall, and F1 scores (87.87%, 86.87%, 84.86%, and 85.85%, respectively; **Figure 2C**). Receiver operating characteristic (ROC) analysis demonstrated strong discriminative performance across most classes, with AUC values exceeding 0.96 (**Figure 2D**). However, performance varied by infection phase, as reflected by class-wise F1 scores (**Figure 2E**). The lowest performance was observed for IAV in the early phase (F1 score 57.14%), followed by SARS-CoV-2 in the early phase (F1 score 80.85%), whereas later-phase classes generally showed higher performance (**Figure 1E**).

Consistent with this, confusion matrix analysis indicated that the early-phase IAV class was most frequently misclassified as normal (**Figure 2F**). These findings suggest that early-phase infection, particularly in the IAV model, exhibits localized and subtle histologic alterations that partially overlap with adjacent phases, thereby reducing classification performance. We also acknowledge that the early phase IAV class was underrepresented (training n=11; evaluation n=3; **Figure 2A**), which likely contributed to the reduced F1 score in addition to subtle early lesions.

Overall, these results indicate that CNN-based histopathologic analysis can distinguish virus type and infection phase once inflammatory lesions are sufficiently established. Given the reduced performance in the early phase, we focused subsequent analyses on the peak-injury and resolution phases, when lesions were clearly established, to characterize time-evolving histologic states within each virus model.

### 3.3 Deep learning–guided lung histopathology reveals alveolar-dominant SARS-CoV-2 lesions and bronchocentric influenza A lesions during the peak-injury phase

**Figure 3:**
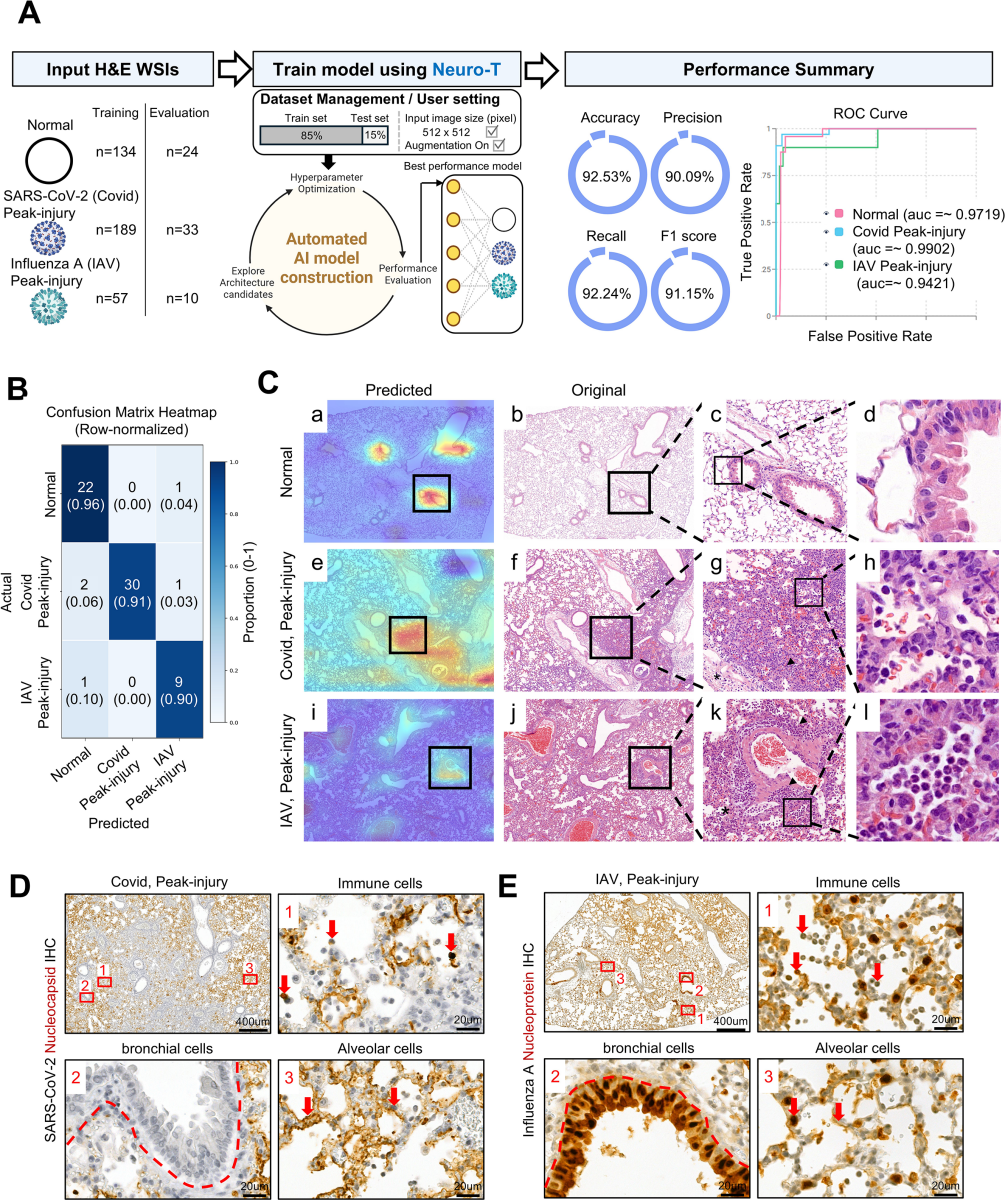
CNN-based pathogen-specific classification of lung histopathology at the peak-injury phase. **(A)** Schematic overview of the CNN-based classification pipeline. Representative lobe-level H&E WSIs from normal control, and from SARS-CoV-2- and influenza A–infected lungs during the peak-injury phase were used for multi-class classification. Extracted image patches were processed through a convolutional neural network, and overall model performance was summarized by accuracy, precision, recall, F1 score, and ROC curve analysis. **(B)** Row-normalized confusion matrix showing classification performance across normal, SARS-CoV-2 (peak-injury phase), and influenza A (peak-injury phase) groups. In each cell, the upper number indicates the number of evaluation images, and the value in parentheses indicates the row-normalized proportion for the corresponding actual class. **(C)** Class activation maps highlighting discriminative regions used by the CNN model for classification. Heatmaps (Predicted) are overlaid on representative H&E images and compared with the corresponding original sections. Boxed regions correspond to areas with high activation in the heatmap, and the enlarged panels display the matched regions in the original H&E images. perivascular edema (asterisks), and inflammatory cell accumulation (arrowheads) are indicated. **(D)** Representative immunohistochemical (IHC) staining for SARS-CoV-2 nucleocapsid protein in lung sections during the peak-injury phase. High-magnification images correspond to boxed areas. Red arrows indicate representative cells corresponding to the labeled compartments (immune cells_box1, bronchiolar cells_box2, and alveolar cells_box3). Scale bars: 400 μm (low magnification) and 20 μm (high magnification). **(E)** Representative IHC staining for influenza A nucleoprotein in lung sections during the peak-injury phase. High-magnification images correspond to boxed areas. Red arrows indicate representative cells corresponding to the labeled compartments (immune cells_box1, bronchiolar cells_box2, and alveolar cells_box3). Scale bars: 400 μm (low magnification) and 20 μm (high magnification).

To assess whether virus-specific histologic patterns could be distinguished during the peak-injury phase, we applied a 3-class CNN classification to digitized lobe-level H&E lung images from normal controls, peak-injury phase SARS-CoV-2 lungs, and peak-injury phase IAV lungs (**Figure 3A**). Because semi-quantitative inflammation scores were broadly comparable between the two infection models during the peak-injury phase, overall inflammatory burden alone was unlikely to explain class separation (**Figure 1G**). Nevertheless, the model showed strong performance, with clear separation among classes on ROC analysis (**Figure 3A**). To further evaluate class-level discrimination, confusion matrix analysis was performed. Misclassification was limited not only between normal and infected lungs but also between the two infected groups, peak-injury phase SARS-CoV-2 and peak-injury phase IAV lungs (**Figure 3B**), indicating that the model captured virus-associated histologic differences beyond simple separation from normal tissue.

CAM further highlighted distinct virus-specific spatial attention patterns during the peak-injury phase (**Figures 3A–C, E, I**). Representative heatmaps paired with original H&E images and high-magnification views demonstrated that model attention consistently overlapped with lesion-enriched regions in a virus-dependent manner (**Figures 3B, C, F, J**). In SARS-CoV-2–infected lungs, model attention was broadly distributed throughout the alveolar parenchyma and preferentially localized to regions showing diffuse interstitial septal thickening and widespread inflammatory cell infiltration (**Figures 3C–G**). Peribronchial and perivascular edema was also observed (**Figures 3C–F**). At higher magnification, SARS-CoV-2 lesions showed mixed inflammatory infiltrates, with relatively greater lymphocytic representation than neutrophilic infiltration (**Figures 3C–H**). In contrast, although IAV-infected lungs also showed attention distributed across the alveolar parenchyma, model attention was relatively more concentrated around bronchiolar structures, particularly in areas with bronchiolar epithelial alterations, dense peribronchial inflammatory cuffing, and patchy perivascular aggregates (**Figures 3C–K**). Peribronchial and perivascular edema was likewise observed in IAV-infected lungs (**Figures 3C–J**). At higher magnification, the inflammatory infiltrate in IAV-infected lungs was composed predominantly of neutrophils (**Figures 3C–l**).

To investigate the cellular basis underlying these distinct lesion patterns, we examined viral localization during the peak-injury phase using immunohistochemistry (**Figures 3D, E**). In SARS-CoV-2–infected lungs, viral antigen was predominantly detected in alveolar epithelial cells and scattered infiltrating immune cells throughout the parenchyma, with minimal involvement of the bronchial epithelium (**Figure 3D**). This alveolar-centered viral localization corresponded to the diffuse parenchymal lesion pattern identified by histopathology and CNN analysis. In contrast, IAV-infected lungs exhibited concentrated viral antigen positivity in bronchiolar epithelial cells, frequently accompanied by dense peribronchial and perivascular inflammatory aggregates, with additional multifocal extension into adjacent alveolar regions (**Figure 3E**). This bronchocentric epithelial tropism provides a cellular basis for the bronchocentric lesion pattern identified by CNN-based spatial analysis.

Collectively, these findings demonstrate that CNN-based histopathologic analysis can distinguish virus-specific lung lesion patterns during the peak-injury phase, even when overall inflammatory severity is broadly comparable, by capturing distinct spatial and structural architectures associated with virus-specific epithelial tropism.

### 3.4 CNN-based analysis resolves virus-specific recovery-phase lung architectures during the resolution phase

**Figure 4:**
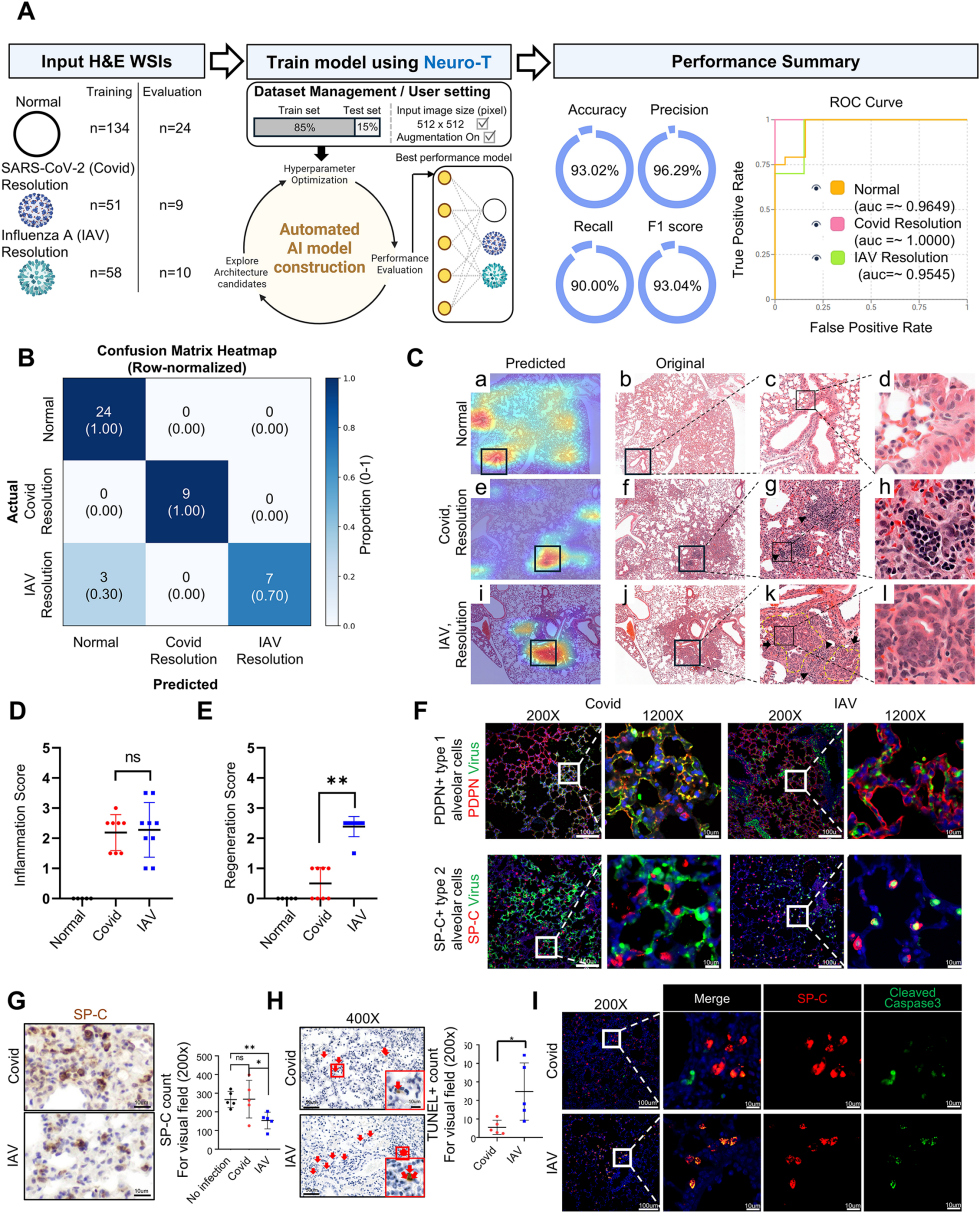
CNN-assisted discrimination of recovery-phase lung lesion patterns during the resolution-phase. **(A)** Schematic overview of the CNN-based classification pipeline for resolution-phase samples. Representative lobe-level H&E WSIs from normal control, and from SARS-CoV-2- and influenza A–infected lungs during the resolution phase were used for multi-class classification. Extracted image patches were processed through a convolutional neural network, and model performance was summarized by accuracy, precision, recall, F1 score, and ROC curve analysis. **(B)** Row-normalized confusion matrix showing classification performance across normal, resolution-phase SARS-CoV-2, and resolution-phase influenza A groups. In each cell, the upper number indicates the number of evaluation images, and the value in parentheses indicates the row-normalized proportion for the corresponding actual class. **(C)** Class activation maps highlighting discriminative regions identified by the CNN model. Heatmaps (Predicted) are overlaid on representative H&E images and compared with the corresponding original sections. Boxed regions correspond to areas with high activation in the heatmap, and enlarged panels display the matched regions in the original H&E images. Inflammatory cell accumulation (arrowheads), and regeneration (arrows, yellow dashed lines) are indicated. **(D)** Semiquantitative inflammation scores of lung sections from normal controls and from SARS-CoV-2- and influenza A–infected mice during the resolution phase. Each dot represents one mouse; group sizes were n = 5 (no infection), n = 8 (SARS-CoV-2, resolution phase), and n = 9 (influenza A, resolution phase). **(E)** Regeneration scores based on histologic evaluation of epithelial repair during the resolution phase. Each dot represents one mouse; group sizes were n = 5 (no infection), n = 8 (SARS-CoV-2, resolution phase), and n = 9 (influenza A, resolution phase). **(F)** Representative immunofluorescence images during the peak-injury phase showing viral nucleocapsid antigen localization relative to alveolar epithelial cell subtypes in SARS-CoV-2– and influenza A–infected lungs. SARS-CoV-2 N-protein signal is shown together with PDPN (AT1 marker) or SP-C (AT2 marker), and influenza A nucleocapsid signal is shown together with PDPN or SP-C, as indicated. Boxed regions are shown at higher magnification. Scale bars: 100 μm (200x) and 10 μm (1200x). **(G)** IHC staining for SP-C in lung sections, with quantification of SP-C–positive cell counts per field (×200). **(H)** Representative TUNEL staining of lungs from SARS-CoV-2– and influenza A–infected mice during the peak-injury phase. Boxed areas are shown at higher magnification. Scale bars: 50 μm (400×) and 10 μm (2500×). Right, quantification of TUNEL^+^ cells per high-power field (HPF, 200×) (n = 5 per group). **(I)** Immunofluorescence staining for SP-C and cleaved caspase-3 in SARS-CoV-2– and influenza A–infected lungs (×200), showing apoptotic alveolar epithelial cells. For panels D and E, data are shown from one representative cohort used for the resolution-phase comparison. For panels F–I, analyses were performed using one representative cohort for each infection group during the peak-injury phase. Where statistical analyses were performed, histopathologic scores were treated as ordinal variables and analyzed using the Kruskal–Wallis test followed by Dunn’s multiple-comparison test. horizontal brackets indicate the group comparisons tested by unpaired two-tailed Student’s t-test. Error bars denote mean ± SD. Significance is indicated as ns (not significant), *P < 0.05, **P < 0.01, and ***P < 0.001.

We next examined whether recovery phase histologic outcomes during the resolution phase could also be distinguished at the tissue level. To this end, we performed a 3-class classification using digitized lobe-level H&E lung images from normal controls, SARS-CoV-2–infected mice during the resolution phase, and IAV–infected mice during the resolution phase, analyzed with Neuro-T (v4.4.1) (**Figure 4A**). The model showed strong overall performance, with an accuracy of 93.02%, precision of 96.29%, recall of 90.00%, and F1 score of 93.04%. ROC analysis further demonstrated strong discriminative performance, with AUC values of 0.9649 for normal, ~1.0000 for SARS-CoV-2 during the resolution phase, and 0.9545 for IAV during the resolution phase (**Figure 4A**). Consistent with this, confusion matrix analysis showed minimal misclassification, including limited cross-confusion between SARS-CoV-2 and IAV resolution phase groups (**Figure 4B**).

CAM further indicated that the model focused on distinct morphologic regions in each group during the resolution phase (**Figures 4A–C, E, I**). In SARS-CoV-2–infected lungs, highlighted regions were generally associated with largely preserved alveolar architecture accompanied by multifocal lymphocytic infiltration patterns (**Figures 4C–F**). The peribronchial and perivascular edema observed during the peak-injury phase was largely resolved during the resolution phase (**Figures 4C–F**). At higher magnification, the residual inflammatory infiltrates in SARS-CoV-2–infected lungs were composed predominantly of densely clustered lymphocytes (**Figures 4C–H**). In contrast, IAV-infected lungs more frequently showed attention over regions with epithelial thickening, multifocal cellular aggregates, and more conspicuous remodeling-associated foci (**Figures 4C–J**). Peribronchial and perivascular edema was likewise markedly reduced or largely absent during the resolution phase in IAV-infected lungs, indicating substantial resolution compared with the peak-injury phase (**Figures 4C–J**). At higher magnification, neutrophils were decreased compared with the peak-injury phase but were still detectable, while lymphocytes were increased, resulting in a more mixed residual inflammatory infiltrate (**Figures 4C–L**).

Histopathologic scoring further supported divergent structural outcomes between the two infections: inflammation scores during the resolution phase were broadly similar between infected groups (**Figure 4E**), whereas regeneration scores were significantly higher in IAV-infected lungs than in SARS-CoV-2–infected lungs (**Figure 4F**). Together, these findings indicate that, despite comparable residual inflammatory burden, the two infections diverged in their resolution-phase lesion patterns, with SARS-CoV-2 lungs showing relatively preserved alveolar architecture and lymphocyte-predominant residual inflammation, whereas IAV lungs showed more prominent repair- and remodeling-associated features together with a more mixed residual inflammatory pattern.

To explain these divergent resolution-phase architectures, we examined epithelial tropism and injury during the peak-injury phase, when viral antigen and epithelial damage are most apparent. Subtype-specific immunofluorescence staining demonstrated that, during the peak-injury phase, IAV antigen was more frequently observed in SP-C^+^ type II alveolar epithelial cells (AT2), whereas SARS-CoV-2 antigen was more often detected in PDPN^+^ type I alveolar epithelial cells (AT1) (**Figure 4G**). Reflecting this preferential localization of IAV antigen to SP-C^+^ AT2 cells, quantification by SP-C immunostaining showed that SP-C^+^ cells were reduced in IAV-infected lungs compared with controls, whereas SP-C^+^ cells were relatively preserved following SARS-CoV-2 infection (**Figure 4H**). In addition, TUNEL analysis and immunofluorescence staining for cleaved caspase-3 with SP-C demonstrated increased apoptosis of SP-C^+^ cells in IAV-infected lungs compared with controls (**Figures 4I, J**). Collectively, these data suggest that preferential AT2 injury, targeting key progenitors for alveolar repair, may contribute to the more prominent regeneration/remodeling signature observed during the resolution phase.

Accordingly, these data provide a cellular correlate for the divergent resolution-phase outcomes: IAV shows preferential AT2 targeting with AT2 loss/apoptosis, consistent with a heightened regeneration/remodeling response, whereas SARS-CoV-2 shows relative sparing of SP-C^+^ AT2 cells with AT1-skewed antigen localization, consistent with a more preserved alveolar architecture and lymphocyte-predominant residual inflammation.

### 3.5 A lung histology scoring guide for preclinical SARS-CoV-2 and influenza A studies informed by distinct histologic trajectories

**Figure 5:**
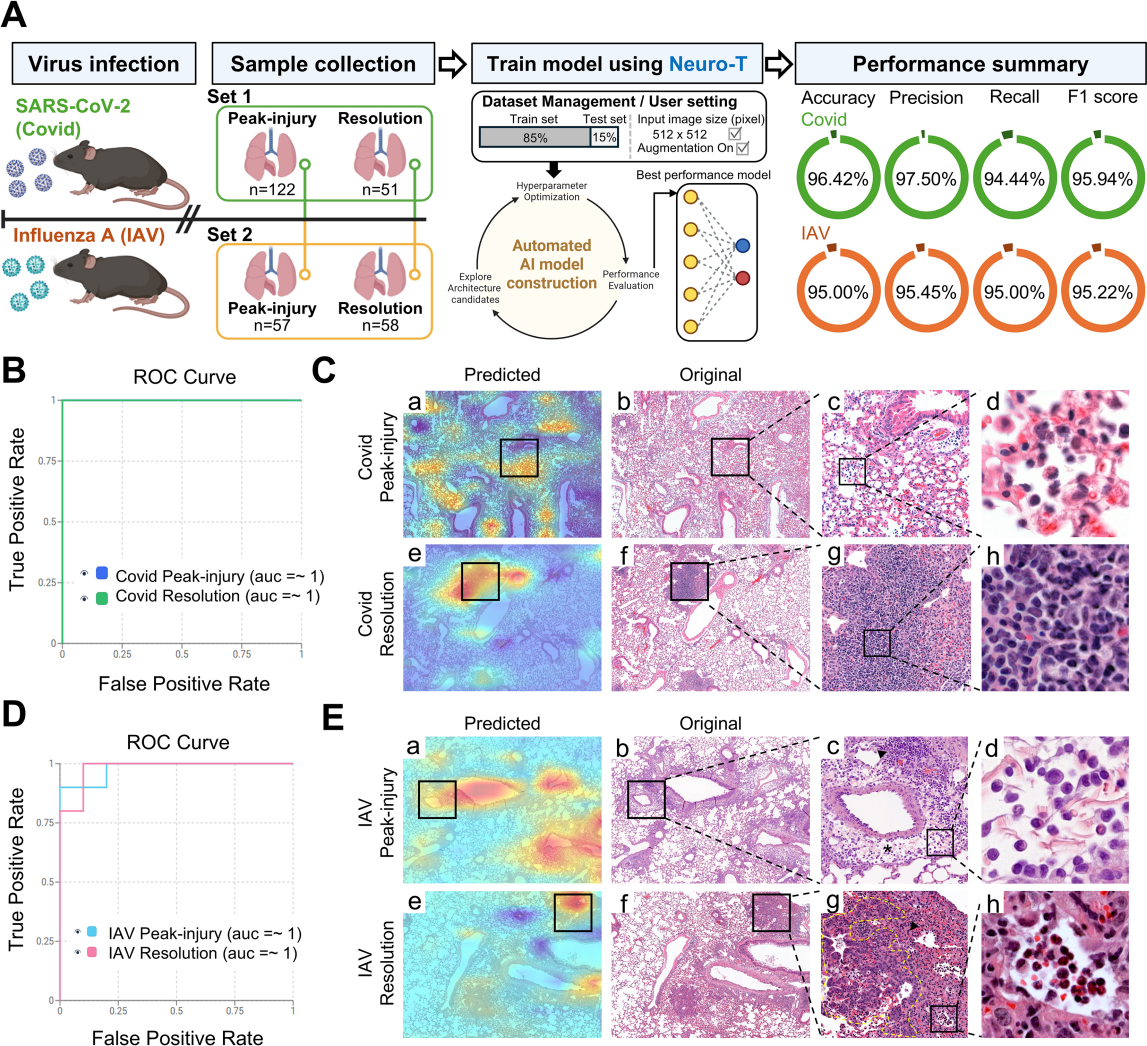
Time-point classification and comparative morphologic features of SARS-CoV-2 and Influenza A infection during the peak-injury and resolution phases. **(A)** Schematic overview of the experimental design and CNN-based classification workflow. Lung tissues were collected from SARS-CoV-2– and influenza A–infected mice during the peak-injury and resolution phases. Representative H&E images were processed through a convolutional neural network to classify infection phase (peak-injury phase vs. resolution phase) within each virus group. Model performance was summarized by accuracy, precision, recall, and F1 score for each pathogen. **(B)** Receiver operating characteristic curve for phase classification (peak-injury phase vs. resolution phase) in SARS-CoV-2–infected lungs. **(C)** Class activation maps for SARS-CoV-2 phase classification. Heatmaps (Predicted) are overlaid on representative H&E images and compared with the corresponding original sections. Boxed regions indicate areas with high activation in the heatmap, and enlarged panels display the matched regions in the original H&E images. **(D)** ROC curve for phase classification (peak-injury phase vs. resolution phase) in influenza A–infected lungs. **(E)** Class activation maps for influenza A phase classification. Heatmaps (Predicted) are overlaid on representative H&E images and compared with the corresponding original sections. Boxed regions correspond to highly discriminative regions identified by the model. Perivascular and interstitial edema (asterisks), inflammatory cell accumulation (arrowheads), and regeneration (yellow dashed lines) are indicated.

Given the virus-specific lesion patterns identified during the peak-injury and resolution phases in **Figures 3**, **4**, we next examined whether temporally distinct histologic states could be resolved within each infection model by directly comparing the peak-injury and resolution phases using CNN-based classification and CAM (**Figure 5A**). Separate binary classifications were performed for SARS-CoV-2 and IAV using digitized lobe-level H&E lung images analyzed with Neuro-T (v4.4).

In SARS-CoV-2 infection, viral antigen was most prominent during the early phase and was markedly reduced thereafter, with near-complete clearance during the resolution phase (**Figure 1I**). In parallel, inflammation scores decreased during the resolution phase compared with the peak-injury phase (**Figure 1G**). Consistent with these changes, CNN-based classification accurately distinguished SARS-CoV-2 lungs in the peak-injury phase from those in the resolution phase (accuracy 96.42%, precision 97.50%, recall 94.44%, and F1 score 95.94%; **Figure 5A**; Green), with ROC analyses supporting robust phase discrimination (**Figure 5B**). CAM analysis further revealed phase-associated differences in the spatial distribution of discriminative regions (**Figure 5C**). During the peak-injury phase, CAM signals were broadly distributed throughout the parenchyma, consistent with diffuse inflammatory involvement (**Figures 5A–C**). During the resolution phase, highlighted regions became more localized and corresponded to residual lesion foci with focal architectural distortion (**Figures 5C–F**). At higher magnification, inflammatory infiltrates showed a time-dependent shift in composition, with substantial neutrophilic involvement during the peak-injury phase and increasing lymphocyte predominance during the resolution phase, when residual inflammation appeared as more densely clustered lymphocytic foci (**Figures 5C, D, G, H**). Together, these results indicate a transition from diffuse parenchymal inflammation during the peak-injury phase to localized residual foci during the resolution phase in SARS-CoV-2 infection.

In IAV infection, viral antigen peaked during the peak-injury phase and showed limited residual positivity during the resolution phase (**Figure 1J**). In contrast to SARS-CoV-2, inflammation scores remained comparably high between the peak-injury and resolution phases (**Figure 1G**). Despite this sustained inflammatory burden, CNN-based classification robustly distinguished IAV lungs in the peak-injury phase from those in the resolution phase (accuracy 95.00%, precision 95.45%, recall 95.00%, and F1 score 95.22%; **Figure 5A**; Orange), and ROC analyses likewise supported phase separation (**Figure 5D**). CAM heatmaps demonstrated phase-associated differences in discriminative regions (**Figure 5E**). During the peak-injury phase, highlighted regions were frequently localized to bronchocentric areas with peribronchial/perivascular inflammatory aggregates and patchy consolidation (**Figures 5A–E**). By the resolution phase, highlighted regions became more focal and were associated with repair/remodeling features, including focal epithelial thickening and persistent architectural alteration (**Figures 5E, F**). At higher magnification, IAV lungs in the peak-injury phase showed neutrophil-predominant infiltrates, whereas IAV lungs in the resolution phase showed reduced but persistent neutrophils with increased lymphocytic representation, indicating a time-dependent shift in inflammatory-cell composition during recovery (**Figures 5C–E, G, H**). Thus, IAV progresses from bronchocentric inflammatory aggregates during the peak-injury phase to focal repair-associated lesions during the resolution phase.

**Figure 6:**
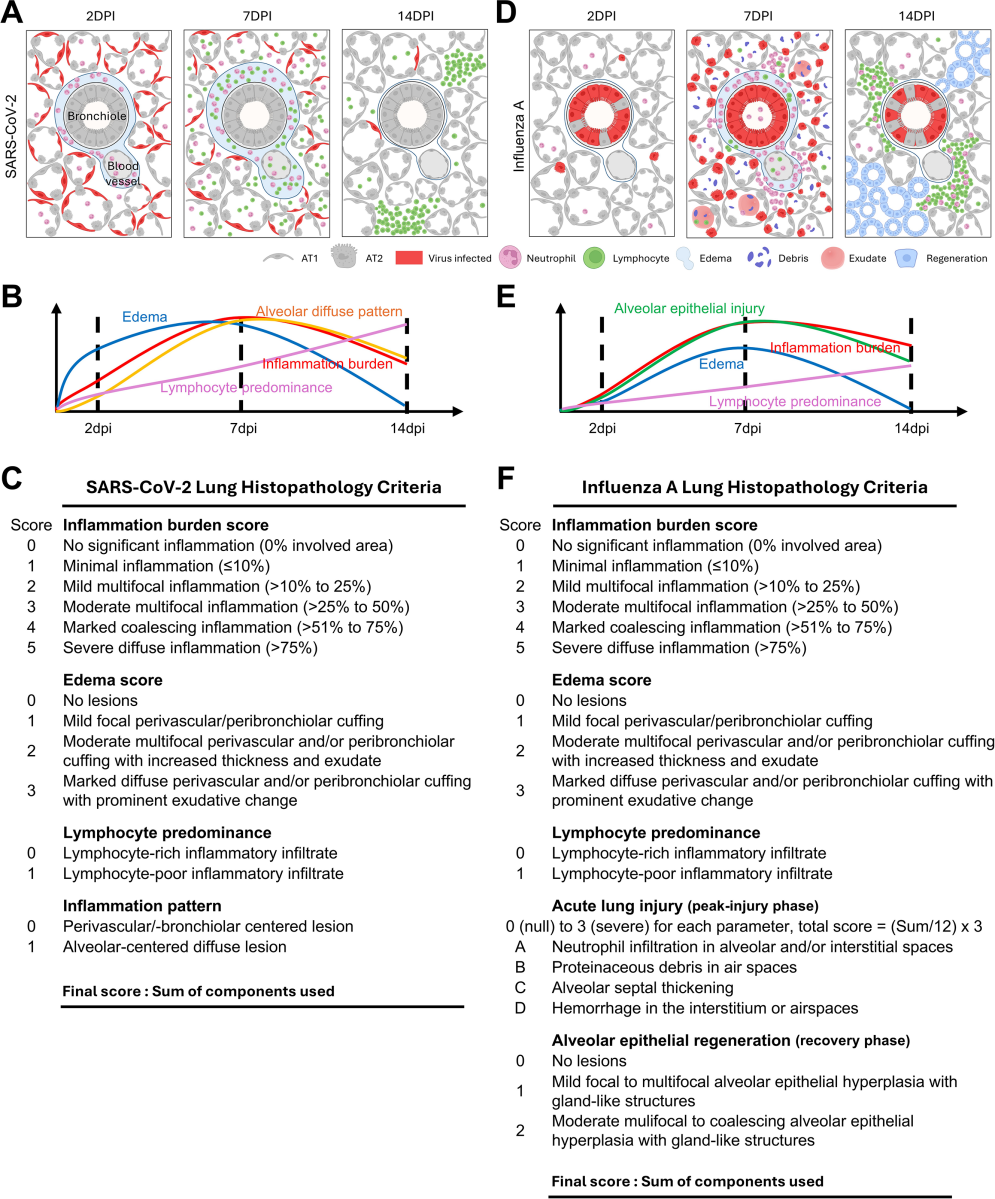
Schematic summary of lung histopathologic progression and scoring system in SARS-CoV-2 and influenza A infection. **(A)** Schematic illustrations of representative phase-specific lung histopathologic features at 2, 7, and 14 days post-infection in SARS-CoV-2 infection, showing alveolar structures, bronchioles, and blood vessels with immune cell infiltration. **(B)** Graphical representation of temporal changes in histopathologic parameters evaluated in SARS-CoV-2 infection, including inflammation burden, edema, inflammation pattern, and lymphocyte predominance. **(C)** Semiquantitative scoring criteria used for lung histopathologic evaluation in SARS-CoV-2 infection. **(D)** Schematic illustrations of representative phase-specific lung histopathologic features at 2, 7, and 14 dpi in influenza A virus infection. **(E)** Graphical representation of temporal changes in histopathologic parameters evaluated in influenza A infection, including inflammation burden, edema, alveolar epithelial injury, and lymphocyte predominance. **(F)** Semiquantitative scoring criteria used for lung histopathologic evaluation in influenza A infection.

Taken together, these analyses demonstrate virus-specific temporal trajectories in lung histopathology. In the SARS-CoV-2 mouse model, infection is initially characterized by widespread alveolar involvement with early edema and neutrophil-dominant inflammation, which progresses to diffuse parenchymal inflammation during the peak-injury phase, despite a relative reduction in viral burden (**Figures 6A, B**). During the resolution phase, lesions become more localized and are characterized by lymphocyte-predominant inflammatory foci (**Figures 6A, B**). Based on these features, the scoring criteria for SARS-CoV-2 prioritize (i) inflammation burden, (ii) edema, (iii) lymphocyte predominance, and (iv) inflammation pattern (localized versus diffuse parenchymal involvement) (**Figure 6C**). Because overt epithelial injury is not a prominent feature in this model, an acute lung injury scoring component was not included.

In contrast, the IAV mouse model shows an initial bronchial epithelial tropism that progressively extends to the alveolar compartment, reaching maximal viral burden during the peak-injury phase (**Figure 6D**). This phase is characterized by prominent neutrophil-dominant inflammation, alveolar epithelial injury, and tissue damage (**Figures 6D, E**). During the resolution phase, although viral burden declines, residual inflammation remains relatively active, with a shift toward lymphocyte predominance accompanied by a prominent regenerative response (**Figures 6D, E**). Accordingly, the IAV scoring system incorporates an acute lung injury component to capture epithelial injury and tissue damage (**Figure 6F**). The acute lung injury score was adapted from established histopathologic scoring systems in experimental lung injury models and modified to reflect the specific features observed in this study (21, 32–34). Atelactasis can also be considered a feature of acute lung injury; however, it was not included as a scoring parameter because lung collapse can be influenced by technical factors during tissue processing, particularly suboptimal lung inflation, thereby limiting its reliability as a reproducible histopathologic metric.

Collectively, this pathogen-resolved scoring framework extends conventional burden-centered grading by incorporating phase-specific lesion distribution, edema, epithelial injury, and repair-associated responses, together with context-dependent changes in inflammatory-cell composition.

## 4 Discussion

In this study, we directly compared lung histopathology between severity-matched SARS-CoV-2 and influenza A virus (IAV) infection models and found that comparable overall inflammatory burden can mask pathogen-specific differences in the spatial organization and qualitative character of lung injury and recovery. Despite similar aggregate inflammation scores, the two viruses differed in lesion topology, epithelial tropism, local inflammatory-cell composition, and the architecture of residual lesions during the resolution phase. SARS-CoV-2 more frequently involved broader interstitial and alveolar compartments during the peak-injury phase and transitioned toward localized residual inflammatory foci, whereas IAV showed more bronchocentric acute lesions followed by more conspicuous remodeling-associated changes during the resolution phase. These distinctions support the view that, for experimental viral pneumonia, lesion distribution and residual structural organization represent key dimensions of interpretation that are not fully captured by burden-based grading alone. This interpretation is consistent with prior pathology literature showing that severe viral pneumonias may share major injury categories while still differing in lesion localization, compartmental involvement, and remodeling context (4, 35).

Beyond these pathogen-specific findings, the broader significance of this study lies in organizing complex viral pneumonia morphology into practical, pathogenesis-informed scoring criteria for preclinical respiratory virus models. Although major histopathologic features of SARS-CoV-2 and IAV infection have been reported previously, there remains a practical need to clarify how these features should be prioritized, organized, and scored when overall inflammatory burden overlaps across pathogens or disease phases. In the present study, similar levels of overall inflammation were associated with distinct spatial and morphologic patterns depending on pathogen and disease phase. During the peak-injury phase, comparable inflammation scores corresponded to alveolar/interstitial-dominant SARS-CoV-2 lesions and bronchocentric IAV lesions. During the resolution phase, despite broadly similar inflammation scores, SARS-CoV-2 lungs showed lymphocyte-predominant residual inflammation, whereas IAV lungs showed more prominent repair- and remodeling-associated features with a mixed residual inflammatory pattern. In this context, CNN/CAM analysis was not used to make infection-phase classification a final endpoint, but rather to provide an objective whole-slide approach for identifying reproducible spatial patterns that may be missed by conventional burden-based scoring. These CNN-highlighted patterns were then interpreted together with expert histopathology, viral antigen localization, and epithelial tropism analysis to inform scoring parameters that were biologically supported, reproducibly assessable, and appropriate for each model. Thus, this study provides a practical bridge between descriptive viral pneumonia pathology and standardized scoring, which may be useful for preclinical vaccine, antiviral, and pathogenesis studies in which histopathology is used to compare disease stage, pathogen-specific tissue responses, and treatment-associated modulation of lung injury.

A biologically relevant distinction in our study was the association between epithelial cell targeting and later repair morphology. Type II alveolar epithelial (AT2) cells are established alveolar progenitor cells that self-renew and generate AT1 cells during alveolar repair, and injury to this population can influence downstream regeneration (36, 37). In our model, preferential IAV involvement of SP-C^+^ cells, together with reduced SP-C^+^ cell abundance and increased apoptosis, was associated with higher regeneration scores and more prominent remodeling during the resolution phase. Although these relationships are correlative, they are biologically compatible with the idea that preferential injury to AT2 cells can shape downstream repair trajectories and may contribute to the more remodeling- associated resolution phenotype observed in IAV.

In addition, across both models, local inflammatory-cell composition shifted with time: neutrophils were more represented during the peak-injury phase, whereas lymphocytes became relatively more represented within residual lesions during the resolution phase, consistent with evolving immune organization during recovery (38–40). Beyond overall inflammation burden, our data indicate that the lymphocyte predominance assessed directly in lung tissue captures a qualitative dimension of viral pneumonia that is not reflected by burden-based scores alone. During the peak-injury phase, lesions in both models were characterized by higher neutrophil representation, whereas during the resolution phase the inflammatory milieu shifted toward relatively increased lymphocytic representation within residual lesions, providing tissue-level context for phase-dependent transitions in inflammatory organization. Importantly, we propose lymphocyte predominance as a practical, phase-sensitive histologic indicator of inflammation quality. This provides a simple framework for distinguishing lesions dominated by acute neutrophilic exudative inflammation from those showing a more lymphocyte-enriched residual or interstitial pattern. Incorporating evaluation of lymphocyte predominance—either through standardized manual counting in predefined regions or through AI-assisted cell composition quantification—may improve pathogen- and phase-informed histopathologic interpretation and help refine scoring systems for preclinical viral pneumonia.

Methodologically, our findings underscore the need for CNN-based whole-slide image analysis models in preclinical and laboratory-animal pathology, where histologic readouts are frequently used as primary efficacy or safety endpoints and where reproducibility across studies, observers, and institutions is critical. Semi-quantitative inflammation grading effectively captures overall inflammatory burden, but conventional burden-based scoring may not fully resolve differences in lesion topology, edema dynamics, residual architectural alteration, or inflammatory organization when severity overlaps between groups (18). Consistent with this, conventional inflammation-burden scores did not significantly distinguish the peak-injury and resolution phases in our dataset, likely due to overlapping levels of inflammatory severity. This limitation highlights the need for more integrative, pattern-aware approaches that incorporate spatial distribution and morphologic context beyond overall burden-based grading. Moreover, preclinical slide review is inherently sensitive to field selection and observer weighting, particularly in heterogeneous viral pneumonia where informative regions may be focal or spatially patterned. By contrast, CNN-highlighted regions enable systematic whole-slide surveying and region prioritization, localizing the morphologic features that most clearly separated pathogens and phases and thereby improving spatially informed interpretation. This is in line with the broader utility of whole-slide deep learning for pathology decision support (25). Taken together, these results support CNN-based analysis as a practical adjunct to standard scoring that can improve scalability and consistency of pathogen- and phase-specific histopathologic interpretation in animal studies, and may extend beyond lesion classification to help refine and inform pathogen- and phase-specific scoring.

Despite these strengths, the translational applicability of the present framework should be interpreted within several important limitations. Although mouse models are useful preclinical systems for analyzing virus- and phase-dependent histopathologic patterns, murine models do not fully recapitulate the anatomical, physiological, and immunologic complexity of human lung disease. In particular, the K18-hACE2 model has inherent limitations, including non-physiologic transgene expression and exaggerated neurotropism relative to human infection (9, 41, 42). In addition, the phase-aware scoring approach proposed here should be interpreted in the context of established principles for valid histopathologic scoring, including biological relevance, consistency, and reproducibility (21). Accordingly, the present CNN framework is more appropriately interpreted as a pathology-assistance tool for standardized preclinical readouts rather than as a model intended for direct clinical application in human disease. Future studies should evaluate whether the morphologic signatures and disease trajectories identified here can be reproduced in larger and more human-relevant animal models, including porcine models, as well as in human lung specimens (32, 34, 43, 44). Such external validation will be essential for determining the broader generalizability and translational utility of this approach (45).

In summary, our data show that SARS-CoV-2 and IAV produce distinct patterns of lung injury and recovery-phase lesion architecture despite similar overall inflammatory severity. These findings support a phase-informed, pathogen-aware histopathology scoring system that extends beyond inflammation burden to incorporate lesion topology/compartmental involvement, residual architectural remodeling, and the qualitative character of inflammation. Such an approach should improve the interpretability and comparability of preclinical viral pneumonia studies across pathogens and phases.

## Data Availability

The raw data supporting the conclusions of this article will be made available by the authors, without undue reservation.

## Supplementary material

Supplementary Table 1 Ablation analysis of train method, input image size and augmentation across classification tasks. Performance of the proprietary CNN models was compared across the classification tasks according to input image size, resize method, and augmentation setting. The table summarizes the comparative results used to determine the final training configuration.

Supplementary Table 2 Training parameters of Neuro-T models for each classification task. The table summarizes the available training details of the best-performing proprietary CNN model for each classification task, including batch size, number of epochs, number of layers, and initial learning rate.
