# Supplementary material for: Deep learning–assisted, pathogenesis-informed lung histopathology scoring in preclinical mouse models of SARS-CoV-2 and influenza A infection
Source: Front Immunol. 2026 May 20;17:1826608. doi: 10.3389/fimmu.2026.1826608 (PMC13231504; doi:10.3389/fimmu.2026.1826608)
Supplement: Supplementary Table 1 — Ablation analysis of train method, input image size and augmentation across classification tasks. Performance of the proprietary CNN models was compared across the classification tasks according to input image size, resize method, and augmentation setting. The table summarizes the comparative results used to determine the final training configuration. [file Table1.docx]

Supplementary Table 1. Ablation analysis of train method, input image size and augmentation across classification tasks

| Classification task | Model name  (Size, Augment) | Input image size  (256, 512, 1024) | Augmentation  (On, Off) | Accuracy | Precision | Recall | F1 score | Selected for final analysis |
| --- | --- | --- | --- | --- | --- | --- | --- | --- |
| 7-class classification; *Figure 2 model* | 512×512, Aug on | 512x512 | On | 87.87 | 86.87 | 84.86 | 85.85 | Selected |
|  | 512×512, Aug off | 512x512 | Off | 79.79 | 86.7 | 70.5 | 77.77 | Not selected |
|  | 256×256, Aug on | 256x256 | On | 77.77 | 79.69 | 69.26 | 74.11 | Not selected |
|  | 1024×1024, Aug on | 1024x1024 | On | 86.86 | 88.24 | 84.35 | 86.25 | Not selected |
| Peak-phase cross-virus classification; *Figure 3 model* | 512×512, Aug on | 512x512 | On | 92.53 | 90.09 | 92.24 | 91.15 | Selected |
|  | 512×512, Aug off | 512x512 | Off | 77.61 | 84.45 | 65.12 | 73.54 | Not selected |
|  | 256×256, Aug on | 256x256 | On | 86.56 | 87.07 | 87.07 | 87.07 | Not selected |
|  | 1024×1024, Aug on | 1024x1024 | On | 91.04 | 93.26 | 90.1 | 91.65 | Not selected |
| Resolution-phase cross-virus classification; *Figure 4 model* | 512×512, Aug on | 512x512 | On | 93.02 | 96.29 | 90 | 93.04 | Selected |
|  | 512×512, Aug off | 512x512 | Off | 88.37 | 94.25 | 82.59 | 88.03 | Not selected |
|  | 256×256, Aug on | 256x256 | On | 90.69 | 95.23 | 86.29 | 90.54 | Not selected |
|  | 1024×1024, Aug on | 1024x1024 | On | 93.02 | 96.29 | 89.25 | 92.64 | Not selected |
| SARS-CoV-2 temporal classification; *Figure 5 model* | 512×512, Aug on | 512x512 | On | 96.42 | 97.5 | 94.44 | 95.94 | Selected |
|  | 512×512, Aug off | 512x512 | Off | 89.28 | 87.5 | 92.1 | 89.74 | Not selected |
|  | 256×256, Aug on | 256x256 | On | 75 | 78.12 | 81.57 | 79.81 | Not selected |
|  | 1024×1024, Aug on | 1024x1024 | On | 92.85 | 95.23 | 88.88 | 91.95 | Not selected |
| IAV temporal classification; *Figure 5 model* | 512×512, Aug on | 512x512 | On | 95 | 95.45 | 95 | 95.22 | Selected |
|  | 512×512, Aug off | 512x512 | Off | 90 | 90 | 90 | 90 | Not selected |
|  | 256×256, Aug on | 256x256 | On | 95 | 95.45 | 95 | 95.22 | Not selected |
|  | 1024×1024, Aug on | 1024x1024 | On | 95 | 95.45 | 95 | 95.22 | Not selected |

Performance of the proprietary CNN models was compared across the classification tasks according to input image size, resize method, and augmentation setting. The table summarizes the comparative results used to determine the final training configuration.

Supplementary Table 2. Training parameters of Neuro-T models for each classification task

| Classification task | Model name  (Size, Augment) | Batch size | Epochs | Number of layers | Initial learning rate |
| --- | --- | --- | --- | --- | --- |
| 7-class classification;  Figure 2 model | 512×512, Aug on | 30 | 162 | 52 | 0.002 |
| Peak-phase cross-virus classification;  Figure 3 model | 512×512, Aug on | 14 | 231 | 184 | 0.0005 |
| Resolution-phase cross-virus classification;  Figure 4 model | 512×512, Aug on | 20 | 231 | 6 | 0.00146 |
| SARS-CoV-2 temporal classification; Figure 5 model | 512×512, Aug on | 30 | 530 | 52 | 0.002 |
| IAV temporal classification;  Figure 5 model | 512×512, Aug on | 30 | 739 | 52 | 0.002 |

The table summarizes the available training details of the best-performing proprietary CNN model for each classification task, including batch size, number of epochs, number of layers, and initial learning rate.
